# Beyond the Surgery: Determinants of Infection, Dislocation, and Revision After Hip Hemiarthroplasty

**DOI:** 10.7759/cureus.97961

**Published:** 2025-11-27

**Authors:** Ercan Bayar, Ahmet Burak Satilmis, Hüseyin Sina Coşkun, Leman Tomak, Yılmaz Tomak

**Affiliations:** 1 Department of Orthopaedics and Traumatology, Ondokuz Mayıs University, Samsun, TUR; 2 Department of Orthopaedics and Traumatology, Taşköprü State Hospital, Kastamonu, TUR; 3 Department of Orthopaedics and Traumatology, Ondokuz Mayıs University Faculty of Medicine, Samsun, TUR; 4 Department of Biostatistics, Ondokuz Mayıs University Faculty of Medicine, Samsun, TUR

**Keywords:** bipolar hemiarthroplasty, complication of treatment, elderly hip fractures, elderly trauma, geriatrics < health occupations

## Abstract

Background

Hip fractures are among the most common causes of morbidity and mortality in the elderly population. Hemiarthroplasty is frequently preferred for the surgical treatment of displaced femoral neck and unstable intertrochanteric fractures in older patients. However, postoperative complications such as infection, dislocation, and prosthesis-related failures remain significant concerns that may necessitate revision surgery and increase mortality rates.

Methods

This retrospective descriptive study included 529 patients (177 males, 352 females; mean age 78.7 ± 8.7 years) who underwent hemiarthroplasty for proximal femoral fractures at a single tertiary center between 2012 and 2023. Demographic characteristics, fracture type, surgical approach, operative time, use of additional fixation materials, hospitalization period, intensive care unit follow-up, and mobilization status were recorded. Postoperative infection, dislocation, and revision rates were analyzed. Statistical comparisons were performed using the chi-square test, Fisher’s exact test, and the Mann-Whitney U test.

Results

Postoperative infection occurred in 35 patients (6.6%), with a significantly higher rate in those operated on via the posterolateral approach and in patients requiring additional fixation materials (p<0.05). Longer hospital stays and delayed mobilization were associated with an increased risk of infection. Dislocation was observed in 18 patients (3.4%), with 72.3% requiring revision surgery. Prolonged operative time was significantly correlated with dislocation and revision rates (p<0.05). Overall, 35 patients (6.6%) underwent revision surgery, most commonly due to infection (45.7%), followed by dislocation (34.2%), aseptic loosening (11.4%), and periprosthetic fracture (8.6%).

Conclusion

Postoperative complications after hemiarthroplasty, particularly infection and dislocation, substantially contribute to morbidity and the need for revision surgery in elderly patients. Strategies such as minimizing the use of additional fixation materials, optimizing early mobilization, reducing hospital stay, and avoiding unnecessary prolongation of surgery may help lower complication and revision rates, thereby improving patient outcomes.

## Introduction

With the aging population and increasing life expectancy, the likelihood of hip fractures increases in patients over 55 years of age [1]. While the annual number of hip fractures worldwide was 1.66 million in 1990, the estimated number of hip fractures and surgeries in 2050 is 6.26 million [2]. Femoral neck fractures constitute 3-5% of all fractures and 57% of hip fractures [3]. These fractures are among the top 10 causes of physical disability in adults worldwide and the second leading cause of hospitalizations in the elderly [4]. Since complications resulting from bedridden conditions after hip fracture increase morbidity and mortality, the goal of treatment in this patient group is to enable early ambulation and restore functional capacity to preoperative levels.

Approximately 98% of hip fractures are treated surgically [5]. A small portion of these patients have a high mortality risk associated with surgery and anesthesia and are considered inoperable [6]. Surgical treatment options include internal fixation, hemiarthroplasty, and total hip arthroplasty. Hemiarthroplasty is a more frequently preferred treatment option, particularly in older patients, due to its reduced bleeding risk, shorter operative time, and ability to facilitate early mobilization. Our clinic has also preferred hemiarthroplasty for unstable fractures in older patients. Complications associated with hemiarthroplasty include infection, dislocation, periprosthetic fracture, acetabular wear, and prosthesis loosening. Prosthesis infection and dislocation are among the most distressing complications, as they can lead to the need for revision surgery, which may negatively impact morbidity and mortality in older patients. Taking the necessary precautions to reduce or prevent complications following hemiarthroplasty will influence mortality and morbidity, and understanding the risk factors contributing to these complications is crucial.

The aim of this study was to retrospectively examine postoperative complications and determinants of infection, dislocation, and revision in patients admitted for hip fractures and treated with hemiarthroplasty, to compare the identified factors with the current literature, and to offer recommendations to reduce complication rates.

## Materials and methods

This retrospective descriptive study included patients who underwent hemiarthroplasty for traumatic proximal femur fractures at the Department of Orthopedics and Traumatology, Ondokuz Mayıs University Faculty of Medicine, between 2012 and 2023. A total of 815 patients were identified using the “hemiarthroplasty” surgery code searched through the hospital information management system (MERSİS).

Inclusion and exclusion criteria for the study

The study included patients who underwent primary hemiarthroplasty for traumatic proximal femur fractures between 2012 and 2023, patients with full access to preoperative and postoperative clinical and radiological data, patients with at least 6 months of follow-up data, and patients aged 55 years and older. A total of 286 patients were excluded from the study if their preoperative and postoperative radiographs were unavailable, if their medical data were missing or deleted from the hospital automation system, if they or their first-degree relatives could not be reached despite telephone contact attempts, if their hip fractures were non-traumatic (pathological fracture, metastasis, primary bone tumor), if they had a history of previous surgery around the hip (e.g., previous hip replacement, internal fixation, osteotomy, etc.), if they had bilateral hip fractures or other concurrent major orthopedic surgery, or if follow-up visits were not available for any reason up to 12 months postoperatively. The study continued with the remaining 529 patients.

Surgical protocol and postoperative approach

The patients underwent necessary examinations and imaging studies upon presentation to the emergency department. Informed consent was obtained from patients who were offered hemiarthroplasty, signed by the patient and their first-degree relatives. First-generation cephalosporin 2 g IV was administered to all patients half an hour before surgery and continued at a 3 × 1 g dose for an average of 2 weeks postoperatively, until the sutures were removed. After completing intraoperative prosthesis trials for each patient, the surgical site was rinsed with diluted baticon, surgical sets were replaced with sterile ones, and the original stem and bipolar heads were applied. Cemented or uncemented prostheses were applied with an average of 10-20 degrees of anteversion and approximately 45 degrees of inclination targets. A Hemovac drain was placed in each patient’s wound site and removed an average of 2 days after surgery. Postoperatively, patients admitted to the ward were ambulated the same day, and those admitted to the intensive care unit were ambulated on the same day as those admitted to the Orthopedics and Traumatology ward and mobilized the following day. Following mobilization, each patient underwent quadriceps exercises as tolerated under the supervision of a physiotherapist. Patients were placed with a pillow between their legs, and their legs were held in an abducted position. To avoid the risk of postoperative dislocation, it was recommended that patients receive assistance for daily needs such as putting on shoes and socks for the first three months and avoid movements that would elevate the knee joint above the hip joint or cause excessive hip flexion.

Findings such as wound drainage, suture dehiscence, increased warmth, and redness at the surgical site, whether during hospitalization or after discharge, were considered surgical site infection (superficial and deep tissue). All patients suspected of infection received early IV antibiotic support and local debridement. Patients who continued to exhibit persistent signs of infection underwent revision surgery. Closed reduction was initially attempted in patients presenting with dislocations. The closed-reduced prostheses were remobilized after a short period of rest. If closed reduction was unsuccessful, patients underwent revision surgery.

Postoperative follow-up included standard radiographs. All patients were recalled for physical examination and radiological evaluation on the 15th day, 1st month, 3rd month, 6th month, 12th month, and annually thereafter.

Data collection and statistical analysis

Demographic characteristics of the patients, such as age and gender, surgical approach type, surgery duration, fracture type, time to surgery, postoperative intensive care follow-up, hospital stay, and postoperative mobilization levels at the 1st and 6th months were evaluated and compared with postoperative infection, postoperative dislocation, and revision surgery history.

Analyses were performed using IBM SPSS version 25 (SPSS Inc., Armonk, NY, IBM Corp., USA). Descriptive statistics included mean, standard deviation, median, minimum, and maximum for continuous data, and frequency and percentage for categorical data. The Kolmogorov-Smirnov test was used to assess normality. The Mann-Whitney U test was used for pairwise comparisons where the normal distribution assumption was not met. Pearson's chi-square and Fisher's exact tests were used to compare categorical data. A p-value <0.050 was considered significant in the interpretation of analyses.

Ethics

The study followed the Declaration of Helsinki. It was conducted with the approval of the Ondokuz Mayıs University Clinical Research Ethics Committee (Decision no: 2024/296; Approval date: 29/07/2024).

## Results

Of the 529 patients included in the study, 177 (33.5%) were male and 352 (66.5%) were female. The overall mean age of these patients was 78.70 ± 8.72 years. The mean age of male patients was 77.84 ± 7.64 years, and the mean age of female patients was 79.13 ± 9.20 years.

The number of patients who experienced deep and superficial surgical site infections in the postoperative period was 35 (6.6%). When comparing the average age of patients who experienced postoperative infection with the average age of patients who did not, no significant relationship was found (p=0.412). Similarly, when comparing the gender distribution of patients who did and did not experience infection, no significant relationship was found (p=0.855).

Of 529 patients, 393 (74.3%) underwent hemiarthroplasty via the posterolateral approach and 136 (25.7%) via the lateral approach. A significant correlation was found between the surgical approach and postoperative infection (p=0.046). The risk of infection was significantly higher in patients who underwent the posterolateral approach (Table 1). No significant correlation was found between the surgical approach and the statistically comparable rates of dislocation (p=0.583) and revision surgery (p=0.427).

**Table 1:** Infection analysis by surgical approach. *Fisher’s exact test. A comparison of paired groups was performed. p < 0.05 was considered significant.

Approach / Infection	Infection	No infection	Total	p*
Posterolateral	31 (7.9%)	362 (92.1%)	393 (100%)	0.046269
Lateral	4 (2.9%)	132 (97.1%)	136 (100%)	-
Total	35 (6.6%)	494 (93.4%)	529 (100%)	-

Univariate and multivariate logistic regression analyses were performed to compare surgical approaches with postoperative infection. The model was found to be borderline significant in the univariate regression analysis (p=0.052), while the multivariate regression analysis found it to be insignificant (p=0.100). According to these analysis results, although the posterolateral approach increased the risk of postoperative infection by 2.8 times (OR=2.83), this was not statistically significant.

The median hospital stay of the 529 patients was 7 (range: 1-61) days, and the mean was 8.45 ± 5.79 days. A significant difference was found when comparing these values with postoperative infection rates (p=0.0066). Patients with infections had longer hospital stays (Table 2). Multivariate logistic regression analysis performed to compare hospital stay duration and postoperative infection showed that the risk of developing infection was significantly higher in patients hospitalized for more than 7 days compared to those hospitalized for less than 7 days (OR=2.33, CI: 1.13-4.81, p=0.023).

**Table 2:** Comparison of hospitalization time and infection. *Mann-Whitney U test. p < 0.05 was considered significant.

Postoperative infection	N	Mean	Std. deviation	Median	Minimum	Maximum	Test statistics	p*
Yes	35	9.43	3.798	9	3	19	U = 6291.50, Z = −2.7120	0.0067
No	494	8.38	5.906	7	1	61		-
Total	529	8.45	5.794	7	1	61		-

When postoperative ICU follow-up of the 529 patients was examined, 203 (38.4%) patients were followed in the ICU, while 326 (61.6%) were transferred to the ward without being followed in the ICU. No significant difference was found when comparing postoperative infection with ICU admission (p=0.720). This indicated that the risk of infection did not increase in patients followed in the ICU after surgery, demonstrating the reliability of our hospital’s ICUs in terms of sterility.

When comparing postoperative infection status with mobilization levels at 1 and 6 months postoperatively, a significantly higher infection rate was found in patients who were immobile at 1 month (p=0.007). Given that patients who were immobile at 1 month postoperatively were more vulnerable, had more comorbidities, and had weaker immune systems, a higher infection rate was expected. Multivariate logistic regression analysis evaluating the effect of 1st-month mobilization status on infection development showed the model to be generally significant (χ²(2)=10.565, p=0.005, OR=2.47). When comparing infection status with mobilization levels at 6 months postoperatively, a statistically significantly lower infection rate was found in patients who were independently mobilized (p=0.010). These findings suggest that the risk of postoperative infection varies depending on the patient’s mobilization status.

The number of patients with dislocations detected in the postoperative period was 18 (3.4%). Of the 18 patients with dislocations, 5 (27.7%) were treated with closed reduction, while the remaining 13 (72.3%) underwent revision surgery. When age and gender analyses were examined for patients with and without dislocations, no significant difference was found between the groups (p=0.863, p=0.800).

The mean time to surgery was 3.00 ± 2.60 days, while the median was 2 (0-24) days. When the time to surgery was compared between patients with and without dislocation, no significant difference was found (p=0.577). When comparing the time to surgery with postoperative infection, the mean time to surgery was 3.71 ± 2.67 days for patients with infection and 2.95 ± 2.59 days for patients without infection. Although the mean time to surgery was longer in patients with infection, the difference was not statistically significant (p=0.099).

Of the 529 patients, 35 (6.6%) underwent revision surgery. Of these 35 patients, 16 (45.7%) underwent revision due to infection, 12 (34.2%) due to dislocation, 3 (8.57%) due to periprosthetic fracture, and 4 (11.4%) due to aseptic loosening. Postoperative infection was the most common reason for revision surgery. Three patients with infection also had a history of dislocation. Revision surgery was performed in patients with postoperative infection who did not respond to IV antibiotics. When comparing the gender distribution of patients who underwent revision surgery with those who did not, no significant relationship was found (p=0.855). No significant relationship was found when comparing the average age of patients who underwent revision surgery with those who did not (p=0.978). The distribution of common causes in patients who underwent revision surgery is shown in Table 3.

**Table 3:** Distribution of revision surgery according to reasons.

Patients who underwent revision	Postoperative infection (Yes)	Postoperative infection (No)	Dislocation (Yes)	Dislocation (No)	Periprosthetic fracture (Yes)	Periprosthetic fracture (No)	Aseptic loosening (Yes)	Aseptic loosening (No)	Total
N	18	17	14	21	3	32	4	31	35
%	51.40%	48.60%	40.00%	60.00%	8.57%	91.43%	11.40%	88.60%	100.00%
Descriptive comparison of revision causes. Normal distribution was assessed. No statistical test was used.									

When operative times were analyzed, the mean operative time was 73.83 ± 27.15 minutes, and the median value was 75 (20-180) minutes. A significant difference was found when operative time was compared with dislocation rates (p=0.012). The risk of dislocation increased significantly with longer operative times (Table 4). Multivariate logistic regression analysis comparing operative time with postoperative infection showed that operative time longer than 75 minutes was a significant risk factor for postoperative dislocation (OR=3.56, 95% CI: 1.31-9.67, p=0.013). No significant difference was found between operative time and postoperative infection rates (p=0.982). When the operative times of patients requiring revision surgery were compared, a significant difference was found (p=0.025). These results indicate that longer operative times increase dislocation rates but do not affect infection rates (Table 5).

**Table 4:** Comparison of surgery time and dislocation. *Mann-Whitney U test. A comparison of paired groups was performed. p < 0.05 was considered significant.

Variable	Dislocation	No dislocation	Total	Test statistics	p
Surgery duration (mean ± SD)	86.11 ± 18.83	73.40 ± 27.31	73.83 ± 27.15	U = 3019.50, Z = −2.506	0.012
Surgery duration (median, min-max)	90 (60-120)	75 (20-180)	75 (20-180)	-	-

**Table 5:** Comparison of surgery duration and revision surgery. *Mann-Whitney U test. p < 0.05 was considered significant.

Revision surgery / Surgery duration	N	Mean	Std. deviation	Median	Minimum	Maximum	Test statistics	p*
Yes	35	82.14	23.524	90	45	120	U = 6709.00, Z = -2.240	0.025
No	494	73.24	27.318	75	20	180		-
Total	529	73.83	27.154	75	20	180		-

Preoperative and postoperative radiographs of the 529 patients were reviewed to determine fracture type. Two separate classifications were made. First, fracture types were divided into four groups based on anatomic location: Type 1: subcapital femoral neck fractures; Type 2: transcervical femoral neck fractures; Type 3: basocervical femoral neck fractures; and Type 4: intertrochanteric femoral fractures. Type 1 fractures were identified in 40 (7.6%) patients, Type 2 fractures in 122 (23.1%), Type 3 fractures in 81 (15.3%), and Type 4 fractures in 286 (54.1%). In the second classification, based on materials used in treatment, 390 (73.7%) patients treated with prosthetic material alone were categorized as Type 1, and 139 (26.3%) patients treated with domino cable, grip plate, or other additional fixation materials were categorized as Type 2.

When postoperative infection was compared with fracture type based on the materials used in treatment, infection rates were significantly higher in patients with additional materials (p<0.001). Multivariate logistic regression analysis comparing fracture types and postoperative infection according to treatment materials showed that the model was generally significant (p<0.001). According to the results, the likelihood of developing infection was 3.6 times higher in patients who required additional fixation materials compared to those who did not (OR=3.645, 95% CI: 1.802-7.373). No significant difference was found when postoperative infection was compared with fracture classification based on anatomic location (p=0.135). According to this result, fracture type itself did not affect infection, but additional materials used in treatment increased infection risk. When patients who underwent revision surgery were compared based on fracture classification according to treatment materials, the revision rate was significantly higher in fracture types requiring additional materials (p=0.004).

## Discussion

Patients who undergo hemiarthroplasty after hip fractures are at risk for both fracture-related and surgery-related complications, particularly with advanced age. Wound infection and dislocation are among the most distressing complications associated with surgery. The high rate of revision surgery required for these complications in older patients further increases mortality and morbidity. Taking the necessary precautions during hemiarthroplasty for hip fractures may reduce infection and dislocation rates, naturally decrease revision surgery rates, and indirectly reduce morbidity and mortality.

The infection rate after hemiarthroplasty is between 0% and 10% [7]. First-generation cephalosporin prophylaxis administered 30 minutes before surgical incision reduces major infections from 5% to 1% and minor infections from 11% to 4% [8]. Postoperative infection has been shown to increase mortality rates and adversely affect morbidity in many studies [9,10]. In our study, postoperative infection was detected in 35 of 529 patients (6.6%). In the literature, this rate was reported as 3.7% by Jalovaara P and Virkkunen H [11] and 4.5% by Tellisi N and Wahab KH [12]. Another study reported that surgical site infection rates after hemiarthroplasty ranged from 1.7% to 7.3% [13]. The infection rates highlighted in the literature and those in our study corroborate each other. Therefore, our study demonstrates that it is comparable to the literature specifically regarding infection. In our study, we examined the mean age and gender distribution of patients who experienced infection in the postoperative period and found no significant relationship between infection and age or gender. Ridgeway S et al. found a significant relationship between increasing age and increasing infection rates [14]. Lau AC et al. found no significant relationship between infection and age or gender [9]. In another study, although the number of postoperative infections was higher in men, no statistically significant difference was found between genders [15].

When comparing postoperative infection rates in patients operated on via the posterolateral and lateral approaches, infection was found to be significantly higher in cases operated via the posterolateral approach. In the multivariate logistic regression analysis comparing surgical approaches with postoperative infection, the posterolateral approach increased the risk of postoperative infection by 2.8 times (OR = 2.83), although this was not statistically significant. There are studies in the literature that found no significant difference in infection rates between the posterolateral and lateral approaches [16]. However, some studies report higher infection rates in patients operated via the posterolateral approach compared with the lateral approach [17]. More studies are needed on this topic.

In our study, no significant difference was found when comparing operative time and postoperative infection rates. There are studies in the literature that have found significantly lower infection rates in patients with shorter operative times [14]. Zajonz D et al. associated early infection after hemiarthroplasty with increased operative time [18]. de Jong L et al. compared cases with surgery times shorter than 45 minutes with cases with surgery times longer than 90 minutes in terms of postoperative infection [19]. It has been emphasized that damage to the soft tissues surrounding the hip increases the risk of infection when the surgery is too short, and that prolonged surgery increases the risk of infection due to increased contamination. It has been stated that surgeries should be kept to an optimal level rather than being either short or long. While there are more studies in the literature supporting the results of our study, there are also studies arguing the opposite. Further research is needed on this topic.

When comparing hospital length of stay with postoperative infection rates, infection was found to be significantly higher in patients with longer hospital stays. Studies in the literature support our findings, emphasizing that infection rates increase significantly with prolonged hospitalization [9,14]. Infections are more common in patients with long hospital stays due to increased exposure to hospital flora and the risk of pressure sores, which may further predispose to surgical site infection. Therefore, ensuring that patients are discharged as early as safely possible is crucial.

In a comparison of patient mobilization levels with postoperative infection, a significant correlation was found between infection and immobility at the first-month follow-up. At the sixth-month follow-up, infection rates were significantly lower in patients who were independently mobilized. Mobilization level is an important parameter when assessing infection risk. Wound-healing problems and early infection signs may contribute to increased immobility at the first month. Studies in the literature report that infection negatively impacts morbidity in patients undergoing hemiarthroplasty, reduces mobilization and rehabilitation, and prolongs hospital stay [9,20].

In our study, no significant difference was found between infection and time to surgery. Some studies, however, indicate that postoperative infection increases with longer time to surgery [9,14]. Waiting time for surgery is not the only risk factor for postoperative infection. According to our results, variables such as length of hospital stay and fracture type also increased the risk of infection, which may explain the discrepancy between our findings and those in the literature.

When comparing postoperative ICU stays with postoperative infection, no significant relationship was found. We did not identify studies in the literature making this direct comparison. However, some studies report that pressure sores are more common in patients with prolonged ICU stays after orthopedic surgery and that these pressure sores may facilitate surgical site infection [20]. In our study, patients who underwent hemiarthroplasty were admitted to intensive care as a precaution, and their average ICU follow-up time was one day. The lack of an increased infection risk in our ICU cohort may be attributable to this short duration.

Our study compared postoperative infection rates based on fracture type. No significant correlation was found when fracture type was classified according to anatomical location. However, when classified according to the materials used in treatment, infection was significantly higher in the group requiring additional fixation materials. Although no comparable study exists in the literature, Schmidt AH and Swiontkowski MF in their study on fracture fixation, noted that increasing the number of materials used facilitates infection by providing additional surfaces for pathogen adhesion and biofilm formation [21]. In our study, additional materials used for fixation beyond the prosthesis increased postoperative infection rates.

The incidence of post-prosthetic dislocation is approximately 1-10% [22]. Excessive anteversion or retroversion of the prosthesis, posterior capsulectomy, or excessive rotation or flexion with the hip adducted after surgery may lead to dislocation. When applying the femoral stem, the prosthesis should be placed at 10°-15° anteversion relative to the femoral diaphysis. Prostheses placed in retroversion carry a risk of dislocation during flexion and internal rotation, while prostheses placed in excessive anteversion carry a risk during extension and external rotation. An oversized prosthetic head may not fit the acetabulum properly, whereas an undersized head may not provide adequate vacuum stability, both contributing to dislocation risk. In our study, dislocation was detected in 18 of 529 patients (3.4%). Of these, 5 (27.7%) were treated with closed reduction, while the remaining 13 (72.3%) underwent revision surgery. Our closed-reduction success rate was 27.7%. Salem KM et al. [23] reported a dislocation rate of 1.76% and a closed-reduction success rate of 23%. Sierra RJ et al. [24] reported a dislocation rate of 2.1% and a closed-reduction success rate of 30%. Although the success rate of closed reduction in first-time dislocations is low both in our study and in the literature, we believe that closed reduction should be attempted initially. If unsuccessful, revision surgery should be considered, and the patient and their relatives should be informed accordingly prior to the procedure.

When comparing dislocation rates by surgical approach type, although the number of dislocations was higher with the posterior approach, no statistically significant difference was found between the lateral and posterior approaches. While some studies found a significantly higher rate of dislocations with the posterolateral approach [16,25], others found no significant difference in dislocation rates between the two approaches [26,27]. There is no clear consensus in the literature on this issue. Depending on the surgical approach, the direction of the dislocation also tends to be the same [28]. Posterior dislocation in the posterolateral approach is associated with forward bending and sitting, i.e., hip hyperflexion and internal rotation, while anterior dislocation in the lateral approach is associated with hyperextension and external rotation, movements that are more difficult to achieve. This may be one reason why dislocations are more common in the posterior approach.

In our study, a significant difference was found between dislocation and operative time, with dislocation being significantly more common in patients with longer operative times. Studies in the literature comparing dislocation and operative time support our findings, showing that the risk of dislocation increases with prolonged operative times [23]. When patients requiring and not requiring revision surgery were compared in terms of operative time, the mean operative time for those who underwent revision surgery was significantly longer than for those who did not. We found no studies in the literature comparing operative time between patients requiring and not requiring revision. In our study, although prolonged operative time increased the risk of dislocation, we did not find an associated increase in infection risk. Infection and dislocation are the most common reasons for revision. Based on the results of our study, the main factor contributing to increased revision rates appears to be the rise in dislocation rates with longer operative times. Further research on this topic is needed.

In our study, 35 of 529 patients (6.6%) underwent revision surgery. Of these, 16 (45.7%) underwent revision due to infection, 12 (34.2%) due to dislocation, 3 (8.57%) due to periprosthetic fracture, and 4 (11.4%) due to aseptic loosening. Femoral fractures may occur during medullary preparation or hip reduction maneuvers. To prevent femoral diaphyseal fractures, care must be taken when reaming the femur, the stem diameter must be matched to the medullary canal, and the reduction maneuver must be performed carefully. The acetabular wear seen with unipolar prosthesis designs contributed to the development of bipolar designs. While radiographic acetabular wear reaches 20% in unipolar prostheses, symptomatic wear is reported in 6-8% [29]. Although bipolar prostheses are thought to reduce acetabular wear, functional outcomes of unipolar and bipolar prostheses are similar [29]. To reduce the risk of acetabular wear, the femoral head diameter should be selected carefully, and the use of excessively large or small modular heads should be avoided. Infection was the most common cause of revision in our study. Frihagen F et al. reported a revision rate of 7.1% in patients undergoing hemiarthroplasty and identified infection as the most common reason for revision, consistent with our findings [30]. de Jong L et al. also reported infection as the leading cause of revision [19]. The results of our study and those in the literature corroborate one another. Although infection is the most common reason for revision, dislocation, aseptic loosening, and periprosthetic fractures are also important causes. Because these complications may require revision surgery, it is appropriate to inform patients and their families preoperatively about these risks.

When comparing the revision rates of patients operated on via the posterolateral and lateral approaches, no significant difference was found. While some studies in the literature report significantly higher revision rates with the posterolateral approach [31], others find no difference in revision rates between the two approaches [27,32]. When comparing the posterolateral and lateral approaches in terms of revision surgeries, there are both supporting and contradicting results in the literature. Further studies on this topic are needed.

When revision surgery was compared with fracture types, the revision rate was found to be significantly higher in fracture types where additional materials were used. In our study, we observed the same pattern when comparing infection with fracture type classification based on the materials used in treatment. Considering that infection is the most common cause of revision, we can assume that using additional materials indirectly increases the rate of revision surgery because it increases infection risk. We did not find such a comparison in the existing literature. Future studies will help clarify this issue.

When comparing fracture types according to anatomic location in patients requiring revision surgery, no significant correlation was found. When examining the need for revision in relation to fracture type, it is also beneficial to evaluate the relationship between fracture types and the reasons for revision. In our study, no significant correlation was found between anatomical fracture classification and dislocation or infection. Such comparisons are not available in the current literature. We hope that our study will contribute to the literature and encourage further research.

This study examined surgical site infection and dislocation, the most distressing complications of hemiarthroplasty, and explored the reasons for requiring revision surgery. These complications are known to negatively impact morbidity. Sexson SB and Lehner JT reported that the mortality rate in patients with postoperative complications was three times higher than in those without complications [33]. Blanco JF et al. compared the effect of dislocation on mortality in patients who underwent hemiarthroplasty after hip fracture and found that one-year mortality was significantly higher in patients with at least one dislocation [34]. Lee J et al. found that infection significantly increased mortality in the first 30 days and within the first year [35].

When we examine the results of our study in detail, we believe that to reduce infection rates, we must optimize mobilization, especially in the first month after surgery, minimize the use of additional materials, and ensure the safe and timely discharge of patients. To reduce dislocation rates, we must avoid unnecessary prolongation of surgery and anticipate that open reduction will be necessary for the majority of patients presenting with dislocations, preparing accordingly. Only through these measures can we reduce the morbidity and mortality associated with complications following hemiarthroplasty.

Limitations and strengths of the study

This study possesses several methodological strengths, including its large sample size, long observation period, and the comprehensive evaluation of multiple perioperative variables such as surgical approach, operative time, mobilization status, and the use of additional fixation materials. The standardized follow-up protocol and clearly defined inclusion-exclusion criteria enhance internal validity and allow meaningful interpretation of postoperative infection, dislocation, and revision patterns.

Nevertheless, the study’s retrospective and single-center design limits causal inference and generalizability. Important confounders, such as comorbidity burden, ASA classification, nutritional status, and surgeon-related factors, could not be fully controlled. The reliance on medical records introduces the possibility of underreporting mild complications, and mobilization status was not assessed using a standardized functional scale. Moreover, the observed association between additional fixation materials and higher infection or revision rates may be influenced by underlying fracture complexity rather than a direct causal effect. These limitations should be considered when interpreting the findings.

## Conclusions

This study demonstrated that postoperative complications following hemiarthroplasty, particularly infection and dislocation, remain significant clinical challenges that increase the risk of revision surgery and negatively impact morbidity in elderly patients with hip fractures. Infection was more frequent in patients who underwent the posterolateral approach, required additional fixation materials, had prolonged hospital stays, or were immobilized in the early postoperative period. Dislocation was strongly associated with longer operative times, and in most cases, revision surgery was required after unsuccessful closed reduction attempts.

To minimize these complications, careful attention should be given to reducing unnecessary operative time, limiting the use of additional fixation materials when possible, and ensuring early mobilization and timely discharge. Optimizing surgical techniques and perioperative management strategies may not only decrease revision rates but also improve survival and functional outcomes in this vulnerable patient population.
